# COVID-19 influenced gut dysbiosis, post-acute sequelae, immune regulation, and therapeutic regimens

**DOI:** 10.3389/fcimb.2024.1384939

**Published:** 2024-05-28

**Authors:** Sterlin T. Raj, Alexander W. Bruce, Muralidharan Anbalagan, Hemalatha Srinivasan, Sasikala Chinnappan, Mogana Rajagopal, Kushagra Khanna, Harish C. Chandramoorthy, Ravishankar Ram Mani

**Affiliations:** ^1^ Department of Molecular Biology, Ekka Diagnostics, Chennai, Tamil Nadu, India; ^2^ Faculty of Science, University of South Bohemia, České Budějovice, Czechia; ^3^ Department of Structural & Cellular Biology, Tulane University School of Medicine, New Orleans, LA, United States; ^4^ School of Life Sciences, B. S. Abdur Rahman Crescent Institute of Science and Technology, Chennai, India; ^5^ Department of Pharmaceutical Biology, Faculty of Pharmaceutical Sciences, University College of Sedaya International UCSI University, Kuala Lumpur, Malaysia; ^6^ Department of Pharmaceutical Technology, Faculty of Pharmaceutical Sciences, UCSI University, Kuala Lumpur, Malaysia; ^7^ Department of Microbiology and Clinical Parasitology, College of Medicine, King Khalid University, Abha, Saudi Arabia; ^8^ Center for Stem Cell Research, College of Medicine, King Khalid University, Abha, Saudi Arabia

**Keywords:** SARS-CoV-2, gut microbiome, immunomodulation, gut dysbiosis, probiotics

## Abstract

The novel coronavirus disease 2019 (COVID-19) pandemic outbreak caused by severe acute respiratory syndrome coronavirus-2 (SARS-CoV-2) has garnered unprecedented global attention. It caused over 2.47 million deaths through various syndromes such as acute respiratory distress, hypercoagulability, and multiple organ failure. The viral invasion proceeds through the ACE2 receptor, expressed in multiple cell types, and in some patients caused serious damage to tissues, organs, immune cells, and the microbes that colonize the gastrointestinal tract (GIT). Some patients who survived the SARS-CoV-2 infection have developed months of persistent long-COVID-19 symptoms or post-acute sequelae of COVID-19 (PASC). Diagnosis of these patients has revealed multiple biological effects, none of which are mutually exclusive. However, the severity of COVID-19 also depends on numerous comorbidities such as obesity, age, diabetes, and hypertension and care must be taken with respect to other multiple morbidities, such as host immunity. Gut microbiota in relation to SARS-CoV-2 immunopathology is considered to evolve COVID-19 progression via mechanisms of biochemical metabolism, exacerbation of inflammation, intestinal mucosal secretion, cytokine storm, and immunity regulation. Therefore, modulation of gut microbiome equilibrium through food supplements and probiotics remains a hot topic of current research and debate. In this review, we discuss the biological complications of the physio-pathological effects of COVID-19 infection, GIT immune response, and therapeutic pharmacological strategies. We also summarize the therapeutic targets of probiotics, their limitations, and the efficacy of preclinical and clinical drugs to effectively inhibit the spread of SARS-CoV-2.

## Introduction

COVID-19 caused by the SARS-CoV-2 virus, is a respiratory infection that originated in Wuhan, China during December 2019. The virus distributed worldwide causing a pandemic outbreak. The infection caused mild to severe hypoxia state lung injury and organ dysfunction, resulting in a very large number of hospitalisations and deaths. After its entry, through respiratory droplets, SARS-CoV-2 mainly attacks the lining of respiratory epithelial cells. When exposed to SARS-CoV-2, the immune system (both innate and adaptive) launches a coordinated defence to identify and eradicate the virus. Moreover, recent studies have highlighted the involvement of gut microbiota in determining the severity/control of COVID-19 disease through the gut to lung and brain linked axis (Zhao et al., 2022). Increasing analyses have demonstrated the involvement of gut-dysbiosis (changes in microbial community) as one of the main factors for gut associated symptoms and COVID-19 infection causes defects in the gut mucosa and inflammation (Cascella et al., 2023). The gut microbial ecosystem is a modifiable environment that determines the control and severity of the disease and is hence a focus for potential therapeutic intervention. Therefore, the interaction between human health with respect to gut microbiota, particularly the immunological responses and its recovery during post COVID-19, is a fascinating and rapidly evolving topic of contemporary debate. Challenges such as the nature of gut-microbiota, individual variability, and the need for robust clinical trials must all be considered to validate potential effective therapies. Research directions range from increasing our understanding of the role of microbes to developing specific microbiota-targeted treatments, whilst also considering a myriad of other health issues. In this context, cutting-edge and insightful research is critical to gaining a deeper knowledge of the specificity of gut-microbiota and their roles in immunological regulation, infection recovery, and health. As our understanding expands, new methods that harnesses the potential of microorganisms in disease regulation could transform future medical practices. However, to achieving this aim will include deciphering complex biological systems, comprehending potential long-term impacts, realizing therapeutic potential, increasing precision medicine, optimizing therapies, improving complete patient care, and guiding public health policy. Thus, strenuous research efforts could collectively reveal the potential of utilizing the gut microbiota to strengthen immunity and effectively battle COVID-19, with far-reaching consequences for clinical care and the larger landscape of infectious diseases. Indeed, the outbreak of COVID-19 has stimulated multiple research avenues, due to its association with numerous cell types and symptoms (Hannoodee and Nasuruddin, 2022) and the persistent disease outcomes of SARS-CoV-2 infection and its associated symptoms have prompted contemporary researchers to comprehensively address the mechanisms of this disease. Consequently, increased research attention has been paid to the GIT manifestation of SARS-CoV-2.

## ACE2 mediated SARS-CoV-2 infection in the gut

**Figure 1:**
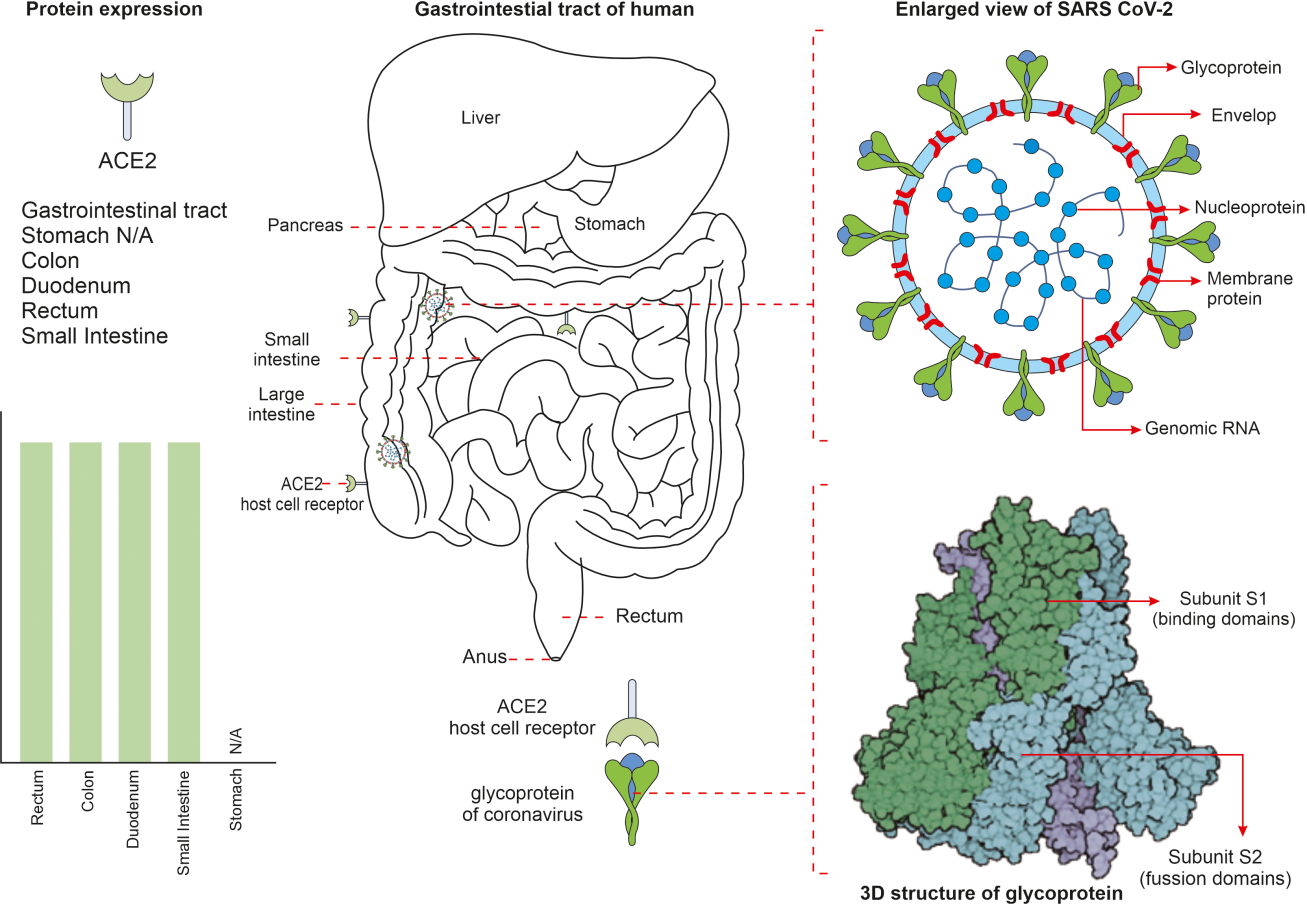
The mechanism behind severe acute respiratory syndrome coronavirus 2 (SARS-CoV-2) cell invasion in some parts of the gut are shown. SARS-CoV-2 virus is surrounded by spike proteins named glycoproteins. The glycoprotein is composed of two functional subunits named S1 and S2, of which the S1 ensures host cell receptor binding and S2 facilitates in fusion of virus into the cell membrane. Once invaded into the human system, the SARS-CoV-2 virus attaches to the angiotensin converting enzyme-2 (ACE2) receptors that are present on cell surfaces of numerous cell types.

The SARS-CoV-2 virus has an outer envelope and a single stranded RNA (ssRNA) genome and infects various human cell types. The virus utilises its spike protein to bind cells of the human respiratory tract and gains entry through the ACE2 receptor. The viral spike protein subunits S1 and S2 assist viral attachment to the ACE2 receptor of the host cell via cell surface S-glycoproteins. Numerous cell types such as goblet cells, ciliated cells, epithelial cells, alveolar cells of the lungs and the GIT, kidney, heart, oesophagus, liver and pancreas express ACE2 receptors (**Figure 1**). ACE2 is,-a homolog of angiotensin-converting enzyme (ACE), that is expressed in a variety of human organs and tissues. It has extensive biological functions and also negatively counteracts numerous other pathways. To date, three Angiotensin-II receptors have been identified, each with similar affinities for Ang-II in the nanomolar range. In the year 2000, two independent research groups discovered ACE2, a homolog of ACE, which can remove the carboxy-terminal phenylalanine in Ang-II to form the heptapeptide angiotensin-(1–7). ACE2 is involved in the uptake of amino acids in the intestinal epithelium. It has been shown that ACE2 is a specific receptor for SARS-CoV-2. Indeed, Zhou et al. (2021) showed SARS-CoV-2 can enter ACE2-expressing cells, but not cells deficient in ACE2 or cells expressing other coronavirus receptors, such as the aminopeptidase N and dipeptidyl peptidase 4 (DPP4); confirming ACE2 is the cell receptor for SARS-CoV-2. Further studies have shown that the binding affinity of the SARS-CoV-2 spike glycoprotein to ACE2 is 10–20 times higher than that of SARS-CoV-2 alone after successful host cell invasion the viral RNA is released. Viral genes are expressed through co-translational and post-translational mechanisms to synthesis several non-structural protein complexes (nsp) such as nsp3, nsp5, and nsp15. Some forms of nsp1–11 and nsp12–16 are synthesized from polyproteins (pp1a) and (pp1ab) and are known to be involved in viral RNA processing, replication and transcriptional functions. Multiple copies of replicated virus trigger immune cells, such as T-helper cells and cytotoxic T cells, to present in the respiratory tract and facilitate in eliminating virus-infected cells. However, COVID-19 is known to not only affect the respiratory system but also other tissues and organs, especially the gut. For example, due to the wide expression of ACE2 and trans-membrane serine protease 2 (TMPRSS2) in intestinal epithelial cells, direct viral attack and tissue damage that can manifest in intestinal disease symptoms and gut dysbiosis can be triggered (Sherif et al., 2023). ACE2 receptors present in gut epithelial cells regulate luminal amino acid levels that in turn contribute to gut homeostasis and drive distinct roles within the gut environment. ACE2 is also known to regulate osmotic maintenance and electrolyte balancing of gut epithelial cells; favouring glucose transport through brush bordered enterocytes. Recent studies have suggested that people susceptible to elevated expression of specific allelic forms of the ACE2 and TMPRSS2 genes are more vulnerable to COVID-19 infection. Symptoms such as indigestion, nausea, vomiting, diarrhoea, and anorexia are commonly reported in COVID-19 patients. Moreover, gastrointestinal imaging reveals thickened bowel walls associated with, abdominal pain, and increased fluid-filled large bowel size, ischemia and pneumatosis. More importantly, such GIT associated symptoms have a direct effect on developing acute respiratory distress syndrome (ARDS) in COVID-19 patients (Kenny et al., 2023). COVID-19 infection also increases the risk of thromboembolic events; including limb venous thrombosis, pulmonary embolism and thromboembolic associated ischemia within the GIT (that is potentially fatal and requires immediate clinical attention) (Wang L et al., 2023).

## Manifestation of innate and adaptive immune cells during SARS-CoV-2

**Figure 2:**
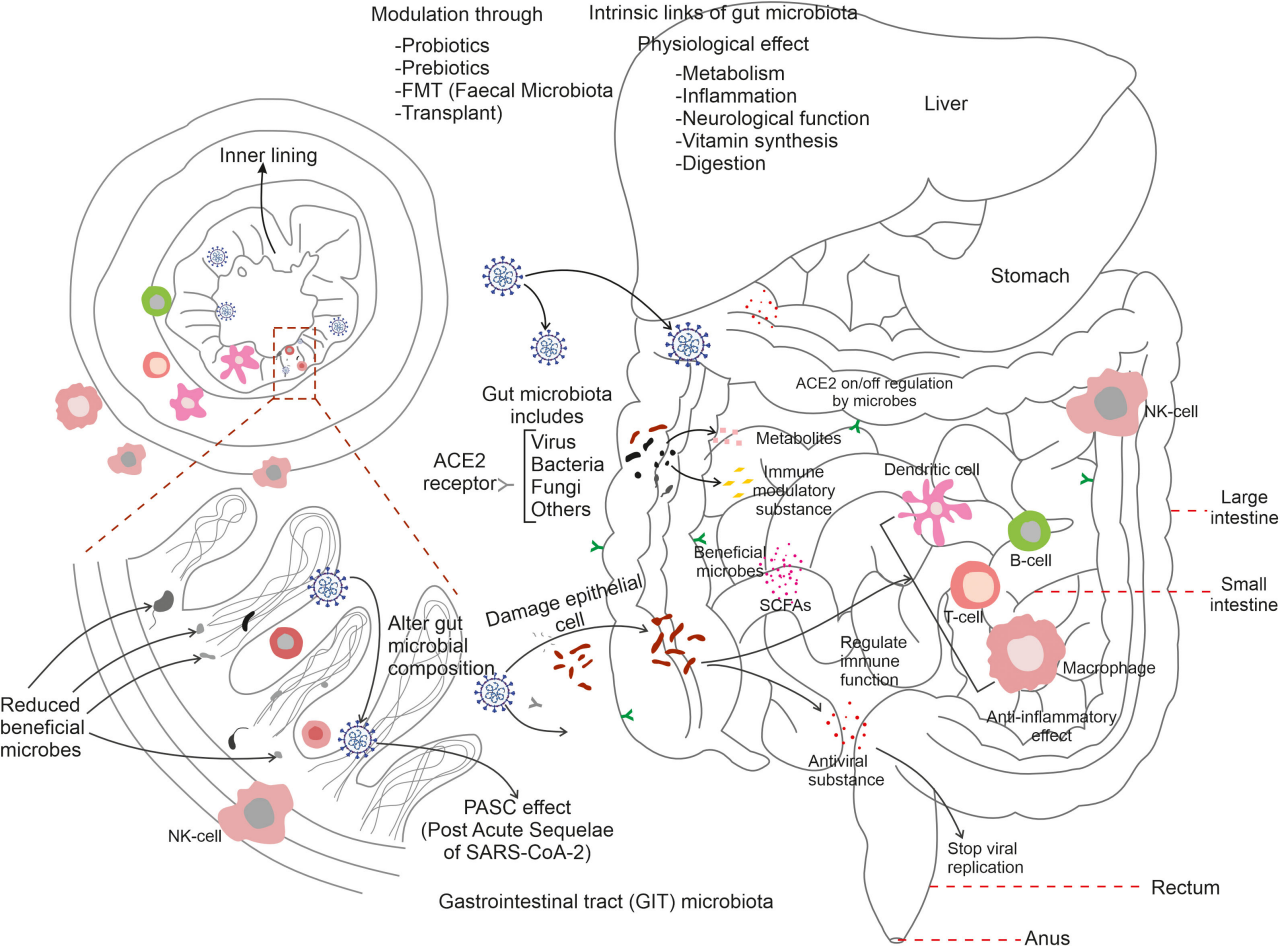
Summarizes the link of gut microbiota with immune cells during COVID-19 infection. SARS-CoV-2 entry activates gut microflora to trigger an immune response through the stimulation of T-cells, B-cells, dendritic cells, and macrophages. Microbial synthesised substances protect neighbouring cells, regulate respiratory cells and the on/off regulation of ACE2 receptors of various tissues. Gut microbes are known to be involved in digestion, inflammation, neurological function, and vitamin synthesis. Reductions in gut-associated-microbes results in serious damage to the inner lining of the mucosal-associated tracts and gut microbiota modulation via probiotics, prebiotics, FMT (faecal microbiota transplant) can restore appropriate gut microbial populations in the post-COVID-19 condition.

Innate immunity is known to be the first-line of defence against infections; whereas adaptive the immune response evolves with time and becomes more pathogen specific. During SARS-CoV-2 entry into intestinal epithelial cells the immune system has been found to be centrally important and is typified by a complicated interplay triggering numerous components and pathways that try to recognise, neutralise, and eliminate the virus. At this stage, innate immune cells such as macrophages and dendritic cells recognise the virus and produce pro-inflammatory cytokines and chemokines via pattern recognition receptors (PRRs) (Chakraborty, and Burns, 2023). Innate immune cells including macrophages and neutrophils recognise the virus (SARS-CoV-2) and generate an inflammatory response. During COVID-19, B-cells produce neutralising antibodies that are capable of binding to the SARS-CoV-2 to mark it for other immune cells to remove. T-cells, including T helper cells and cytotoxic T-cells, assist to recognise and kill virus-infected cells, thus limiting viral propagation. However, the immune response to COVID-19 can become dysregulated, resulting in excessive synthesis and release of pro-inflammatory cytokines; a condition known as a ‘cytokine storm’ resulting in increased inflammation and tissue damage. Cytokine storms are a reflection of severe COVID-19 infection that further results in the development of ARDS and other consequences (Montazersaheb et al., 2022). The immune system generates an immunological memory after the original infection that provides long-term protection against reinfection. When exposed to the virus again, B and T-cells that have already encountered the virus can develop a faster and more efficient immune response (Hung et al., 2021). This memory response serves as a key step in understanding COVID-19 immunity and vaccine development (**Figure 2**). Owing to suboptimal immune function, certain populations such as the elderly, those with underlying health issues, and immune compromised individuals, are highly susceptible to the severity of COVID-19 disease symptoms (Ray et al., 2023). Furthermore, individual variability in immune responses may also lead to variances in disease severity and outcomes. It is noteworthy that upon cellular entry of SARS-CoV-2 through the ACE2 receptor, macrophages serve as the first line defence to limit viral replication. The pro-inflammatory activity of macrophages results through expression of inflammatory cytokines such as IL-1β, IL-6 and the chemokine CXCL10. COVID-19 patients exhibited a defective dendritic cell (DC) response. Conventional DCs (cDCs) from patients with severe COVID-19 exhibited decreased IFN signatures and MHC II expression, contrary to pro-inflammatory pathways. This eventually results the down-regulation of MHC II regulators such as RFX5, RFXANK and CIITA in critical cases. Similarly natural killer cells (NK) exhibit an exhausted phenomenon in COVID-19 patients through expression of inhibitory markers such as NKG2A (Chakraborty et al., 2022). As an important effector molecule, IFN-α suppresses IFN-γ production by NK cells. In regard to the adaptive immune response, CD4^+^ T-cell simultaneously exhibit both highly activated and exhausted stages in patients with severe COVID-19 (Adamo et al., 2021). Thus, SARS-CoV-2 CD4^+^ T-cells are associated with ameliorated COVID-19 disease severity, and the induction of SARS-CoV-2 specific CD4^+^ T-cells is correlated with rapid decreases in viral load. Higher levels of CD4+ T-cells, particularly naive CD4^+^ T-cells, reduces critical illness during COVID-19 infection. In addition, a disproportionality of T-helper type 1 and type 2 (Th-1 & Th-2) cells proportions are found in COVID-19, patients. For example, a higher proportion of Th-17 is observed compared to the levels of Th-1. Thus, a higher proportion of senescent (PDI^+^/ICOS^-^) Th-2 cells are observed in dead patients compared to survivors (Gil-Etayo et al., 2021). When evaluating the relationship of infection categories and deaths, it is noteworthy that Th-2 cells remain a significant risk factor associated with COVID-19 deaths. Indeed, Th-2 cell counts are overall balanced at the stage of COVID-19 recovery and have been observed to later decrease (Fathi et al., 2020). It is suggested that altering the environment of CD4+ cells might skew Th-17 promotion, thereby exacerbating inflammation (Choto et al., 2019; De Biasi et al., 2020); thus, marking Th-17 as a potential therapeutic target. Indeed, inhibitors such as arginase-1 (Arg-1), reactive oxygen species (ROS), and NO synthase (NOS), produced by myeloid cells under oxidative stress, can reverse Th-17 expression via reducing formation of cytokine storms (Parackova et al., 2021). Another mechanism known to maintain immune homeostasis is mediated by regulatory T-cells (Tregs). In ARDS patients, the proportion of both CD4+ and CD8+ Tregs is increased and variation in the amount of Treg cells is observed in mild COVID-19 patients compared to those which were hospitalized. Moreover, Treg targeted therapies have been successfully administrated in disease suppression. The severity of SARS-CoV-2 infection may also been determined by specific CD8^+^ T-cell responses during the acute phase of infection. Several studies suggest that CD8+ T-cells reduce the potential for cytokine production in COVID-19 patients (Mazzoni et al., 2020). Indeed, in the acute phase of COVID-19 infection, CD8^+^ T-cells activate markers such as CD38 and HLA-DR and secrete cytotoxic molecules such as perforins and granzyme B (Sekine et al., 2020) and in the later stages they help reduce viral load and the onset of disease progression (McMahan et al., 2021). During SARS-CoV-2 infection, antibody producing naive B cells and existing B-cells secrete immuno-globulins, manifest by the appearance of serum IgG, IgM and IgA within two weeks of the onset of symptoms (Iyer et al., 2020). Following the onset of COVID-19 infection, the activated specific antibody IgG, neutralizing plasma, memory B and T cells persist for at least 3 months. In addition, the IgG memory B-cell (MBCs) levels increase over time (Rodda et al., 2021). Thus, the adaptive immune response may also be important for controlling SARS-CoV-2 infections. Understanding how COVID-19 affects the immune system is critical for creating effective treatments, vaccines, and preventative approaches. On-going research on understanding the SARS-CoV-2 elicited immune-response is important and is focused, for example, on identifying biomarkers of disease development and treatments that control the immune response to avoid the most severe and deleterious consequences (Hannoodee and Nasuruddin, 2022).

## Regulation of gut-microbiota and immune function

A highly complex and specific population of GIT microbes has evolved to have important roles within the context of several immune responses. The human gut accommodates trillions of microorganisms, which are collectively known as the gut microbiome. They are known to be an essential component of the maintenance of human health and exhibit significant roles in many areas of physiology, including immunological function. The immune system and the gut microbiota have a dynamic and mutually beneficial relationship. Consequently, some significant points emphasising the role of gut microbiota in immune function are illustrated here. The human gut-microbiota is thought to evolve from childhood and plays a critical role in the development and maturation of the immune system. This interaction of immune cells and the gut microbiome establishes a wide immune tolerance and balances both pro-inflammatory and anti-inflammatory responses (Zhang et al., 2023). Thus ensuring, appropriate immune response to infections via preventing excessive inflammation, control of immunological homeostasis, prevention of autoimmune disease, and inflammatory illnesses. GALT (gut-associated lymphoid tissue) is a immunological tissue present in the intestinal mucosa. It is highly populated with immune cells and displays an intimate relationship with gut-microbes (Abo-Shaban et al., 2023). Antigens and microbial components in the human gut encourage the production and activation of immune cells in the GALT, making surveillance and defence against infections immunologically more effective. The gut microbiota contributes a physical barrier in the intestine epithelium; thus preventing the invasion of dangerous bacteria and toxins into the bloodstream. It protects against infections and reduces the risk of systemic inflammation by modulating the tight junctions between neighbouring intestinal cells, and pathogenic microorganisms. The microbial community in the gut creates a wide range of metabolites and immune-modulatory substances that interact with immune cells. Short chain fatty acids (SCFAs), for example, offer anti-inflammatory properties that aid the control of immune cell function. Other microbial products, and metabolites can also alter immune responses and modulate immunity. The influence of the gut microbiota extend beyond the gut by communicating with other organs and tissues to further modulate systemic immune response (Campbell et al., 2023). Understanding the complex links between the gut microbiota and the immune system is a rapidly growing field of research. It is known that modulation of immune responses via diet; probiotics, prebiotics, and faecal microbiota transplantation (FMT) harbour the potential to treat, if not to cure, immunological related changes and gut dysbiosis.

## GIT act as an alternative route of SARS CoV-2

SARS-CoV-2 infection contributes to a wide range of interactions with the human physiology. Following invasion into the GIT, the virus affects the intestinal mucus secreting cells, epithelial cells and potentially affects lamina propria immune cells. Rather than a purely pathogenic effect, the SARS-CoV-2 virus can elicit changes in immuno-related phenotypes GIT infection can be diagnosed through faecal sample analysis in COVID-19 patients, as, viral particles (containing detectable RNA) are able to transit the GIT and are release via the gut-faecal route. Virus particles can also be detected in various other parts of the human GIT; including the duodenum, stomach, and rectum; a viral expressed proteins have also been identified in the cell cytoplasm of duodenal and rectal glandular epithelial cells (Kruglikov et al., 2020). The overall distribution of virus throughout the alimentary canal is however thought to be driven by the respiratory system. However, it is unclear if cellular debris from the lungs contribute. The SARS CoV-2 virus is known to replicate in intestinal cells and to synthesize viral toxins that facilitate cellular changes in the gut and drive disease symptoms (Lamers et al., 2020). Faecal examination of COVID-19 patients reveals the presence of virus for more than 47 days and 16.5 days for sputum specimens. The possible link between the lung and the GIT have been identified in the pathogenesis of SARS-CoV-2 disease. It is well established that the interaction of gut microbiota influences immune response, therefore altering its mobilisation, maturation, and expression (Wang L. et al., 2023). Understanding how COVID-19 is associated with the gut microbiota and the way it alters the immune response to the virus is important for furthering our understanding of the disease progression, its severity, and potential therapeutic approaches. Therefore, the identification of possible biomarkers for disease prognosis is helpful to the development of new therapeutic methods (Hung et al., 2021; Müller and Di Benedetto, 2023).

## Diversity and composition of the gut microbiota

**Figure 3:**
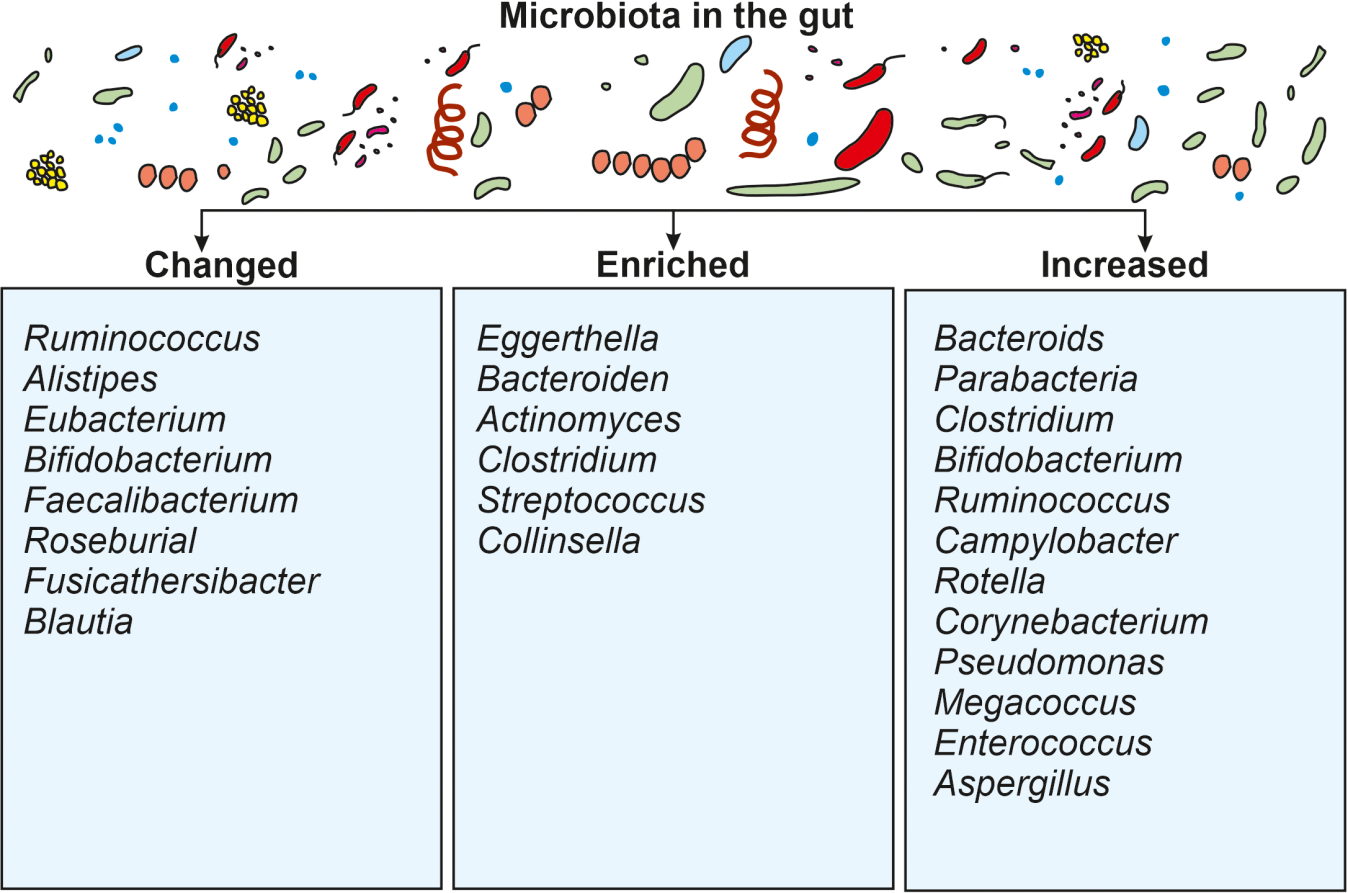
Summarizes changes associated with gut microbiota during SARS-CoV-2 infection.

The gut microbiota is a diverse population of bacteria, fungi, and other microbes found within the digestive tract, particularly the large intestine, that play important roles in regulating the immune system, digestion, vitamin synthesis, and maintaining numerous other physiological and pathophysiological processes. For example, the gut microbiota regulates immune disorders, bowel syndrome, homeostasis, blood pressure, and chronic kidney diseases. Although COVID-19 is largely associated with lung sickness caused by SARS-CoV-2, the virus is expected to move from the lungs into the GIT via the blood and to influence immune response in the gut through a microbial mediated mechanism. The gut colonized microbiota maintain a symbiotic relationship with their host environment by contributing to good health and the elimination of toxins. During COVID-19, the activation of immune cells, such as neutrophils and lymphocytes, result in a drastic reduction of gut microbiota and the secretion of enhanced levels of mucosa. Such disruption in the gut environment reduces the permeability of intestinal cell substances and prevents enterocyte function (Toor et al., 2019). The most common microbial genera that are changed during COVID-19 belong to Ruminococcus, Alistipes, Eubacterium, Bifidobacterium, Faecalibacterium, Roseburia, Fusicathenibacter, and Blautia, as well as the enrichment of Eggerthella, Bacteroides, Actinomyces, Clostridium, Streptococcus, Rothia, and Collinsella. Such changed gut environments induce poor prognosis in patients and realign the microbial ratios (**Figure 3**). Microbiota such as Bacteroides, Parabacteria, Clostridium, Bifidobacterium, Ruminococcus, Campylobacter, Rotella, Corynebacterium, Pseudomonas, Megacoccus, Enterococcus, Aspergillus, Roseburia, Eubacterium, Lachnospira, and Faecalibacterium, are known to be decreased during COVID-19 infection. Reductions in the beneficial bacteria Bifidobacterium are known to increase the invasion of potentially dangerous opportunistic pathogens. Fungal species such as Ascomycota phylum are known to be relatively abundant in COVID-19 patients when compared with non-infected individuals (Fakharian et al., 2023). Therefore, COVID-19 patients frequently demonstrate changes in the relative abundance of specific bacterial taxa. Beneficial bacteria such as Bifidobacterium, Faecalibacterium, and Lactobacillus are found to be less abundant in some studies (Fakharian et al., 2023). Whilst some potentially hazardous bacteria or opportunistic pathogens, such as Streptococcus, Collinsella and Enterococcus may be increased in abundance. A comparative summary of the microbiota of COVID-19 patients during gut dysbiosis is shown in **Table 1**. Such dysregulated microbial composition can influence the severity of COVID-19 cases. In general, similar patterns in altered gut microbiota have been observed in various studies, although some specific findings may differ across individual investigations (SeyedAlinaghi et al., 2023).

**Table 1:** Depicts the range of microbial species in COIVD-19 patients during gut dysbiosis.

COVID-19 Patients	Range	Control group	Range
***Cyanobacteria***	High	*Cyanobacteria*	High
***Lentispaerae***	High	*SHA-109*	High
***Tenericutes***	Moderately–high	*Bacteroidetes*	Moderately–high
***Proteobacteria***	Low	*Verrucomocrobia*	Average
***Bacteroidetes***	Moderate	*Proteobacteria*	Average
***Deferribacteres***	Low	*Chloroflexi*	Low
***Verrucomicrobia***	Moderate	*Actinobacteria*	Average
***Actinobacteria***	High	*Firmicutes*	Average
***Fusobacteria***	High	*Tenericutes*	Moderate
***Unknown phylum***	High	*Euryanchaeota*	Moderately–high
***Firmicutes***	High	*Fusobacteria*	Moderate–high

**Figure 4:**
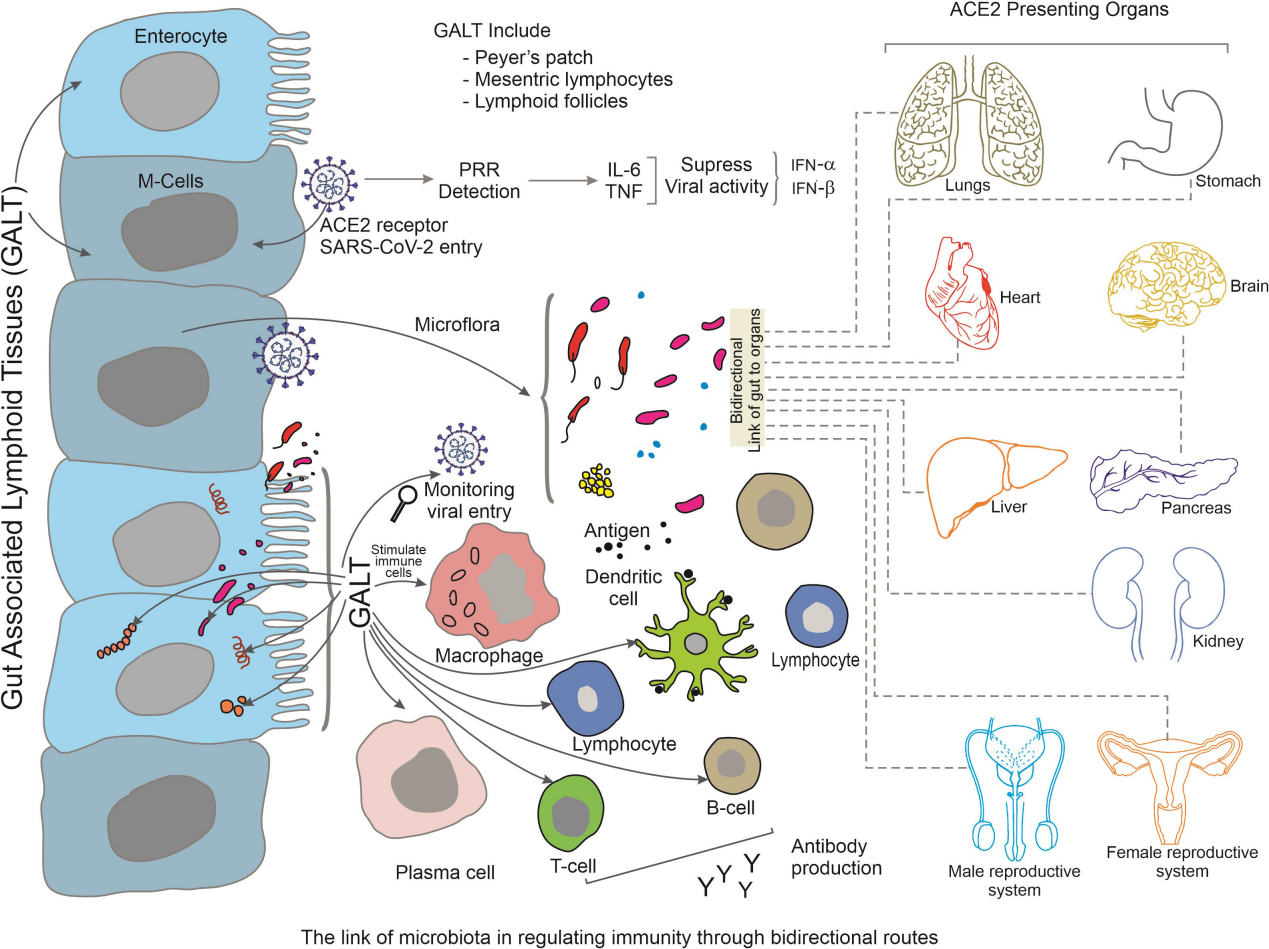
Summarizes the bidirectional link between gut-associated lymphoid tissues (GALT) and other tissues during SARS-CoV-2 infection. Various tissues of GALT, such as Peyer’s patch, mesenteric lymphocytes and lymphoid follicles stimulate immune-activated cells along with the activation of gut microbiota. The activated immune cells migrate to distant organs and maintain regulation of increased immune response via a bidirectional-link-axis. Organs that present ACE2 receptors are highlighted here.

GALT is a lymphoid structure found in the GIT that comprises Peyer’s patches, mesenteric lymph nodes, and isolated lymphoid follicles. In the context of this article, some significant components of GALT are expanded here. GALT acts as a GIT surveillance system, monitoring and responding to infections that enter via the oral route, including SARS-CoV-2. It contains dendritic and macrophages immune cells that recognise viral antigens and generate immunological responses (Park et al., 2023). GALT plays an important role in the initiation of adaptive immunological responses against SARS-CoV-2. Viral antigens are collected by antigen-presenting cells in the GALT, that then migrate to secondary lymphoid organs (such as lymph nodes) to present the antigens to B and T cells. This process results in the production of virus-specific antibodies and also the activation of T lymphocytes capable of attacking infected cells. Thus, GALT aids in the maintenance of immunological tolerance to antigens obtained from food and commensal microorganisms in the gut. Immunological homeostasis is dependent on the balance of immunological responses against infections and tolerance to innocuous antigens. Immune tolerance dysregulation in GALT can contribute to immune-mediated diseases and may influence the severity and course of COVID-19 (Aggarwal et al., 2023). Therefore, GALT is known to have a close association with gut microbiota and displays significant interactions via multiple mechanisms. The development and function of GALT are regulated by the gut microbiota, and GALT in turn affects the composition and function of the gut microbiota. Therefore, changes in the gut microbiota during COVID-19 may have an impact on immunological responses mediated by GALT. Immune responses triggered in GALT can have systemic consequences. For example, activated immune cells originating from GALT, can migrate to other mucosal locations, including the respiratory tract, where they can aid in local immunological responses against SARS-CoV-2. GALT can also modulate the production of cytokines and other immunological molecules that circulate throughout the body and influence the overall immune response (**Figure 4**). Understanding the role of GALT in COVID-19 is critical, particularly in regard to its interaction with the GIT, the immune system, and viral infection. Thus more study is required to fully understand GALT function and its unique contributions to the immune response against SARS-CoV-2 infection and its implications for disease outcomes and future treatment methods (Lionakis et al., 2023).

## Dysregulated immune response affects GIT

The composition of GIT microbiota alters through the increased severity of COVID-19 infections via a mechanism of dysbiosis. This adversely affects the host immune response and host inflammation during disease progression. Commensal and pathogenic microbes are important sources of microbial-associated molecular patterns (MAMPs) and pathogen-associated molecular patterns (PAMPs) (Dhar and Mohanty, 2020). Direct damage to intestinal cells and GIT microbes enhances intestinal permeability and the transfer of microbial mediated signaling molecules into the circulatory system (Uzzan et al., 2020). Pattern recognizing receptors (PRRs) detect the synthesis of microbial derived compounds, leading to immune responses. Moreover, breaches to the integrity of the intestine permit the opportunistic invasion of pathogens into the circulatory system and can result in systemic inflammation. Additionally, this negatively influences the intestinal equilibrium between commensal microbes and host immunity. After SARS-CoV-2 invasion of intestinal cells, innate immune cells such as dendritic cells and macrophages detect and bind viral derived PAMPs that result in the release of pro-inflammatory cytokines that in turn can cause GIT dysbiosis (Katz-Agranov and Zandman-Goddard, 2021). Various immumo-regulatory signaling molecules secreted by *Bacteroids, Bifidobacteria* and *Lactobacillus* regulate innate immune cell metabolism and activities. Thus, the balance of intestinal health and homeostasis is in turn regulated by proinflammatory cells such as Th-17 and Tregs (Dhar and Mohanty, 2020). However, an abundance of SARS-CoV-2 in the GIT provokes the enhanced growth of opportunistic pathogens such as *Burkholderia*, and *Enterococcus faecalis* that leads to T-cell mediated immunity suppression (Zhou et al., 2021). Additionally, during the onset of COVID-19, organisms such as *Blautia obeum, Coprococcus catus*, and *C. comes* can facilitate an increase in the proportion of other specific immune cells types, such as the CD3^+^ T-cells, CD4^+^ T-cells, and CD8^+^ T-cells (Xu et al., 2022). Similarly, opportunistic bacteria such as *E. faecalis*, and *B. contaminans* negatively affect the levels of CD8^+^ T-cells, CD3^+^ T-cells and CD3^+^ T-cells (Sun et al., 2022). SARS-CoV-2 can also induce early neutralising antibody responses that mediate viral destruction. Dysregulated immune responses associated with SARS-CoV-2 infection-induced cytokine storms have been reported in severe COVID-19 cases and result in excessive inflammation and tissue damage. Several factors, including viral load, host immunological factors, and overexpression of pro-inflammatory and anti-inflammatory signals, are known to contribute to this dysregulation (Mohandas et al., 2023; Yuksel et al., 2023). An increased and uncontrolled production of cytokines such as interleukin-6 (IL-6), interleukin-1 (IL-1), and tumour necrosis factor-alpha (TNF) are reported in severe COVID-19 patients. This cytokine storm has the potential to cause extensive inflammation, tissue dysfunction, and organ failure. The cumulative activation of various immune cells, including macrophages, monocytes, neutrophils, and T cells, is frequently related to the cytokine storms observed in severe COVID-19 patients. Moreover, activated immune cells produce more pro-inflammatory cytokines, thereby continuing the inflammatory cascade and enhancing the immunological response. For example, IL1β, IL4, IL5, IL6, G-CSF, granulocyte-macrophage colony stimulating factor (GM-CSF), interferon-γ, IL2, IL10, IL-12/23, IL13, IL15, IL17A, MCP-1, MIP-1β, MIP-1α, sCD40L, TGFα, TNFα, VEGF A, and IL18 are all upregulated in the digestive tissues of rhesus monkeys after SARS CoV-2 infection. As the infection progresses, enhanced levels of inflammatory cytokines are induced in the gut space. Subsequently, numerous anti-inflammatory cytokines such as G-CSF, IL4, IL6, IL13, IL18, MIP-1β, and TNFα are increasingly expressed in the gastrointestinal compartment. This induces pulmonary-derived CC chemokine receptor 9 positive CD4^+^ T cells that are increased after viral infection. Effector CD4^+^ T cells are critical for the development of intestinal mucosal immunity and chronic enteritis, and CCR9 is a chemokine receptor necessary for CD4^+^ T cell entry into the small intestine (Hermens and Kesmir, 2023). Such cells express the C-C motif of the chemokine ligand 25 (CCL25) that can lead to the induction of intestinal immune damage and GIT symptoms. Moreover, in Rhesus monkeys the increased expression of CD68 in the rectum and duodenum has been observed in the initial stages of COVID-19 infection that then reduces to normal levels.

## Susceptibility of the lungs by the immune response of the gut

The connection between the gut and lung axis plays a vital role in the pathology of SARS-CoV-2 infection. The gut microbiota has a major role in regulating good health and immune regulation. Gut dysbiosis through viral infection, such as in COVID-19, stimulates circulatory pro-inflammatory cytokines that alter the composition of the gut microbiome and results in epithelial damage and GIT disease symptoms. Numerous inflammatory cytokines are detected during the initial stage of gut infection in rhesus monkeys. In the later stages of gastric SARS -CoV-2 infection, anti-inflammatory or protective cytokines, including G-CSF, interferon-γ, IL2, IL4, IL10, and MIP-1α, are found to increase in the lung, while inflammatory cytokines such as IL1β, IL1rα, IL5, IL6, IL15, and IL17A are decreased. Thus, as a response to intestinal inflammation, cytokines can also enter the lungs via the blood stream and affect pulmonary immune responses and inflammation (Boechat et al., 2021). In turn, this can dysregulate the gut environment and facilitate the expression of ACE2 expression in the gut, making it more susceptible to the increasing the effects of the SARS-CoV-2 infection. Thus this feedback loop between the gut and the lungs can accelerate and worsen the prognosis of COVID-19 patients.

## Microbial metabolites in immunological modulation

Research on the exact roles of microbial metabolites in immune modulation during COVID-19 is relatively sparse but quickly evolving. However, some general insights have helped to explain how microbial metabolites are related to the elicited immune response and continue to advance. Metabolites secreted by microbes such as short chain fatty acids (SCFAs) and certain indole derivatives are linked to immune regulation and anti-inflammatory properties. An uncontrolled and dysregulate immune response adds severity to COVID-19 and these metabolites may participate in the modulation of immunological response, reducing excessive inflammation and tissue damage (Tieu et al., 2023; Wang M. et al., 2023). Some alpha bacterial strains such as Catenibacterium, Ruminococcus, and Eubacterium produce abundant SCFAs, compared with other delta strains. Strains such as Oscillospirales, Faecalibacterium, and Catenibacterium also produced SCFAs; mainly butyric acid as a response to COVID-19 infection. Stool analysis reports reduced levels of lactic acid and propionic acid in a comparison of COVID-19 infected and healthy individuals. However, the levels of faecal calprotectin (FCP) in both control and infected groups is not elevated; indicating FCP levels are not affected by the associated GIT symptoms. Additionally, metabolomic analysis of COVID-19 patients indicate reduced synthesis of L-isoleucine, decreased indole 3-propionate, and tryptophan metabolites in the severe disease condition (Proal and VanElzakker, 2021). The expression of Zonulin, a protein that regulates tight junction formation in the GIT, is reported to increase the levels of the inflammation marker IL6 and is associated with impaired intestinal barrier function in COVID-19 patients; that also exhibit an increased rate of mortality (Giron et al., 2021). Although GIT composition can also be altered via other lifestyle-based mechanisms, such as smoking, hypertension, and body mass index (BMI), COVID-19 has a marked and explanatory potential to alter the genus and species levels of the gut microbiota. Around 156 species (*P* <.05, FDR < 0.16) of microbes are noted to be significantly changed during COVID-19. It is reported that 10% of 55 COVID-19 implicated microbes can be influenced by background factors such as smoking, antibiotic usage, and dyslipidaemia (Ghimire et al., 2020). Gas and liquid chromatography/mass spectrometry based faecal sample analyses of more than 150 faecal metabolites have identified numerous COVID-19 infection associated alterations. Amino acids are the most notable faecal metabolites. Some of these such as glutamine, proline, phenylalanine, tyrosine, threonine, glycine, tryptophan, aspartic acid, leucin, and valine interact with the GIT. Metabolites that regulate carbohydrate metabolism, such as maltose, sucrose, glutaric acid, SCFAs (acetate and butyrate), glyoxylic acid, xylobiose, N-acetylmannosamine, and isomaltose are significantly depleted in COVID-19 patients (Wang et al., 2020). Neurotransmitters, such as γ-aminobutyric acid, dopamine, and serotonin and other co-factors required for neurotransmitter synthesis, including pyridoxine and pyridoxal 5’-phosphate (vitamin B6)’ were also depleted during COVID-19 infection; moreover, these are positively correlated with observed GIT enriched microbes. Apart from carbohydrates, amino acids, and neurotransmitter-related metabolites, SCFAs including acetate, propionate, butyrate are significantly reduced; again positively correlating with the confirmed communal GIT bacteria (Ghimire et al., 2020). Significantly, still numerous other metabolites such as serotonin, lactulose, nicotinic acid, 3-hydroxybutyric acid, homoserine, and glucosamine are depleted in severe COVID-19 cases. It is also clear that altered microbial metabolites correlate with changes in the functional levels of certain cellular pathways. For instance, the GIT associated degradation pathway of branched chain amino acid (BCAA) positively correlated with COVID-19. Moreover, in relation to the glutathione metabolism pathway, depletion of spermidine, putrescine, ornithine, and glycine are reported in COVID-19 patients. Similarly, pyridoxal 5’-phosphate and pyridoxine levels, related to vitamin B6 metabolism, are also notably depleted. It is also clear that the observed levels of amino acids and many microbial metabolites display significant correlations with the elevated presence of inflammatory cytokines.

## Comorbidities

Certain metabolic disorders, such as obesity and diabetes, are linked to altered gut microbiota and microbial metabolite synthesis. These disorders are known risk indicators associated developing severe COVID-19 (Ananya et al., 2021; Li et al., 2023). Understanding how metabolites interact with the induced immune response in various comorbidities could lead to enhanced understanding of COVID-19 disease outcomes. Interventions, such as the use of prebiotics (substances that promote the growth of beneficial gut bacteria) and probiotics (living beneficial bacteria) may alter microbial metabolite synthesis. These therapies harbour the potential to modify induced immunological responses that in turn may aid the treatment of COVID-19. Although such links are conceivable and supported by existing information related to microbial metabolite production and immune modulation, determining the direct influence of individual metabolites on COVID-19 progression requires careful scientific inquiry. This research is on-going, and will likely provide greater insight into the complex relationships between the gut microbiota, microbial metabolites, and the immunological responses to COVID-19 (Troisi, 2023).

## Metabolic implications of the gut microbial environment

Compositional changes in the gut microbiota are associated with metabolism, including food metabolism and metabolite synthesis. COVID-19 has been linked to metabolic abnormalities such as changes in glucose metabolism and lipid profiles. Understanding the effect of the gut microbiota in association with the metabolism of food on COVID-19 patients can provide additional insight into the disease’s metabolic implications. Accumulating evidence linking COVID-19 must be further explored to confirm the actual causality, underlying mechanisms, and associated therapeutic consequences (Davis et al., 2023). Moreover, the study of the gut microbiota in the context of COVID-19 may have consequences for illness prognosis, identification of biomarkers, and the development of specific treatment methods to lessen disease impact. The significance of gut microbiota in respiratory viral infections, such as those caused by viruses like influenza, respiratory syncytial virus (RSV), and SARS-CoV-2, has been the subject of much recent attention. While gut microbes have historically been connected with gastrointestinal health, new evidence suggests that they also play important roles in regulating immune response, modulation, and respiratory health via the regulation of immune cell formation and maturation and increases in the synthesis of immune-modulating chemicals (Bernard-Raichon et al., 2022). An appropriate composition of the gut microbiome is essential for maintaining an appropriate immune response to respiratory viral infections. The stomach and the lungs are linked by multiple communication routes, known as the ‘gut-lung-axis’. Thus, in relating to immune cell biology the regulation of gut microbiota has an impact on respiratory health by modifying immunological responses, generating compounds that alter lung function, and impacting the integrity of the respiratory epithelium. Therefore, alteration to the original gut microbiota composition impacts lung health and its susceptibility to respiratory virus infections (Bernard-Raichon et al., 2022). Certain gut microbial components have been demonstrated to have antiviral properties. Specific gut bacteria can create antimicrobial peptides that hinder the replication of respiratory viruses. Furthermore, gut microbiota can boost the production of antiviral immune molecules, for example interferons, that are important in fighting viral infections. Equally, dysbiosis can result in chronic low-grade inflammation. Such systemic inflammation can harm the respiratory system and impede the ability of immune cells to fight viral infections. Hence, the regulation of gut microbiota aids immune responses and the maintenance of immunological homeostasis; critical for reducing inflammation during respiratory viral infections (Harper et al., 2021; Portincasa et al., 2022). Microorganisms in the gut are also important in the metabolism of food components such as fibre and complex carbohydrates. SCFAs that are produced by gut microbes have been demonstrated to alter immune response and to affect respiratory health. SCFAs can affect immune cell function and assist in the preservation of respiratory epithelium integrity, regulating susceptibility to respiratory viral infections (Liu et al., 2021).

## Gut microbiota and possible therapeutic targets

Gut associated microbiota have garnered a lot of interest as a potential therapeutic targets for immunological regulation during COVID-19. Such approaches seek to take advantage of the complex connections between gut bacteria and the immune system, in order to modify immune responses and thereby potentially assist beneficial disease outcomes. Promoting the population of beneficial bacteria in the gut could enhance the production of anti-inflammatory compounds that aid the regulation of an appropriate COVID-19-related inflammatory response (Anshory et al., 2023). Therefore, a healthy gut comprised of appropriate microbes seems to be essential in maintaining immunological tolerance and the prevention of autoimmune attacks. Indeed, the gut microbiota could help the immune system discriminate between self and non-self-antigens, thus lowering the likelihood of autoimmunity. Regulation of the ACE2 receptor, that the SARS-CoV-2 virus utilises to enter host cells, may be influenced by specific gut flora. Modulating the gut microbiota may have an effect on ACE2 expression that may affect viral cellular entry and thus infection. The impacts of gut microbiota are not limited to the gut and it can also influence systemic immune responses. Therefore modulating gut microbiota may have far-reaching implications on immune function that may be relevant for the systemic immunological dysregulation observed in severe COVID-19 cases. A person’s gut microbiota is unique (Zhang et al., 2023). Therefore, custom tailoring of therapies to an individual’s specific gut microbiota composition could improve immune regulation. As such modulating the gut flora may be used to supplement existing COVID-19 treatment methods; for example, as a multi-pronged therapy used in combination with antiviral medications and other immune-modulating therapies (Tomkinson et al., 2023). While the prospect of addressing the gut microbiota for immune regulation in COVID-19 is exciting, further research is needed to fully understand the mechanisms, benefits, and potential limitations and concerns. Furthermore, therapies targeted at altering the gut microbiota must be treated with caution because the microbiota is complex and has consequences for many other aspects of human health. Any therapeutic interventions should be supported by solid scientific evidence, well-designed clinical trials, and expert opinion.

The existence of immunological memory, including memory B cells and T cells, suggests that people who have recovered from COVID-19 may be immune to re-infection. Re-infections have been observed, but in comparison to original primary infections, they are relatively uncommon. The amount and duration of protective immunity against reinfection is still being studied and may differ between individuals (Yamamoto et al., 2021). Some studies report residual immunological activation, inflammation, or changes in immune cell subsets in some individuals post-recovery (Abbasi et al., 2022). Activated T-cells and B-cells and humoral immunity during COVID-19 play important roles in the antibody synthesis that targets the spike protein of the virus envelop; also engaging in preventing the binding domain of the spike protein to the epithelial cells of the gut. This indeed safeguards B-cells from reinfection. Although vaccines play a role in viral control, the exact underlying process in relation to the spike protein remains elusive. However, the oral intake of viral vaccines facilitates strong antibody production; such as IgA, IgG and T-helper cells 1, 17 (Th1/Th17) that impedes viral RNA replication in the infected cells (Choto et al., 2019). The effectiveness of viral vaccines can be determined by faecal analysis of the microbial population. Similarly, the presence of antibodies can also be determined via blood serum analysis. The development of vaccines against SARS CoV-2 has indeed provided an important breakthrough in recent years; especially as they are easy to store, administer, are cost effective and biofriendly (Opsteen et al., 2023; Vojdani et al., 2023).

## Interaction of gut microbiota and COVID-19 vaccines

The efficacy of COVID-19 vaccines and their long-term effects are still not fully understood. Numerous studies utilizing animal models indicate variation in GIT microbial composition that correlate/regulate the efficacy of vaccines; suggesting COVID-19 vaccines may have long-term effects (**Table 2**). The ‘Omicron’ variant of SARS-CoV-2 virus, has been found to be less susceptible to COVID-19 vaccine-induced-immunity (Samavati and Uhal, 2020); as the S-glycoprotein associated mutation affects its transmission ability, culminating in immune escape. Although full COVID-19 vaccines do affect some viral variants, such as the Alpha, Beta and Gamma (α, β, γ) variants, booster doses are thought to affect the Omicron variant but at an efficacy that is noted to be less than against the other previously emergent variants of the SARS-CoV-2 virus.

**Table 2:** Illustrates a list of worldwide approved COVID-19 vaccines.

Name of the vaccine	Production	Types of the vaccine	Approved countries	Clinical trials	WHO grants
**COVOVAX**	Serum Institute of India	Protein based	6	9	Approved
**Covishield**	Serum Institute of India	Non-replicating viral vector	49	7	Approved
**Zifvax**	Anhui Zhifei Longcom	Protein based	4	21	Not Approved
**Noora vaccine**	Bagheiat-allah University of Medical Sciences	Protein based	1	3	Not Approved
**Corbevax**	Biological E Limited	Protein based	2	7	Not Approved
**Abdala**	Centre for Genetics and Biotechnology (GICB)	Protein based	6	5	Not Approved
**Recombinant SARS-CoV-2 Vaccine**	National Vaccine and Serum Institute	Protein based	1	3	Not Approved
**Nuvaxoid**	Novavax	Protein based	40	22	Approved
**IndoVac**	PT Bio Farma	Protein based	1	4	Not Approved
**SpikoGen**	Vaxine/CinnaGen Co.	Protein based	1	8	Not Approved
**Razi Cov Pars**	Razi Vaccine and Serum Research Institute	Protein based	1	5	Not Approved
**SKYCovione**	SK Bioscience Co Ltd	Protein based	1	7	Not Approved
**SpikoGen**	Vaxine/CinnaGen Co.	Protein based	1	8	Not Approved
**Covifenz**	Medicago	RNA	1	6	Not Approved
**Comirnaty (BNT1622b2)**	BioNTech	RNA	149	100	Approved
**Covaxin**	Bharat Biotech	Inactivated Virus	14	16	Approved
**KoviVac**	Chumakov Center	Inactivated Virus	3	3	Not Approved
**QazVac**	Research Institute for Biological Safety Problems (RIBSP)	Inactivated Virus	2	7	Not Approved
**COVIran Barekat**	Shifa Pharmed Industrial Co	Inactivated Virus	1	6	Not Approved
**Corona Vac**	Sinovac	Inactivated Virus	56	3	Not Approved

## Clinical studies on gut microbiota

The role of gut microbial populations has been considered in the formulation of vaccine regimes. This is because dysbiosis may impair vaccination efficacy by affecting the immunological milieu and how the immune system reacts to vaccine antigens (Huang et al., 2023; Mańkowska-Wierzbicka et al., 2023). Dysbiosis causes systemic repercussions that migrate beyond the gut environment and cause infection in other organs. Obesity, metabolic syndrome, cardiovascular disease, and neuro-inflammation have all been connected to immunological dysfunction resulting from an unbalanced gut microbiota. It is important to highlight that the interaction between gut dysbiosis, the immune system, and specific infections is complicated and not yet entirely understood. Several clinical studies (**Table 3**) have provided evidence of the link between the influence of gut microflora and COVID-19 vaccines, probiotics, prebiotics, antibodies, and antibiotics (Baradaran Ghavami et al., 2021). A recent trial study demonstrated the response of probiotic Loigolactobacillus coryniformis K8 (LCK8) treatment within health workers (HCWs) who had COVID-19 (confirmed using a periodic PCR (polymerase chain reaction) test). Over 250 voluntary HCWs of 20 years of age and above were administrated with LCK8 for two months; via a daily oral dose of 2x10^9^ CFU (colony-forming unit). The HCWs group who received vaccination had SARS-CoV-2 IgG antibody in the serum and over the course of LCK8 treatment, IgG levels were observed to be higher when compared with the control group. Additionally, it was notable that those patients who consumed probiotics prior to the COVID-19 vaccination, displayed fewer disease associated effects (of any kind) indicating a role in the appropriate maintenance of immune protection (Hussain et al., 2021; Zhang et al., 2023).

**Table 3:** Potential effect of dysregulated gut microbiota during COVID-19 progression.

Gut Microbiota	Potential effect on Immune cells	Outcome	Relative abundance	References
***Acinetobacter, Rhodococcus***	Inversely correlate with CD3, CD4, CD45, haemoglobins and RBC	Promote COVID-19 severity	Upregulated	Wu et al., 2021a
***Bacteroides, Veillonella***	Positively correlate with CD3, haemoglobins and RBC	Lessen COVID-19 severity	Downregulated	Wu et al., 2021a
***Enterococcus***	Positively correlate with the plasma concentration of carbon dioxide	Lessen COVID-19 severity	Downregulated	Wu et al., 2021a
***Eubacterium dolichum, Prevotella copri***	Positively correlate with SARS-CoV-2 viral load	Promote COVID-19 severity	Upregulated	Wu et al., 2021b
***Alistipes, Bi (30) fidobacterium, Clostridium citroniae, Dialister*,** ***Haemophilus, Haemophilus parainfluenzae, Ruminococcus*,** ***Streptococcus anginosus***	Inversely correlate with SARS-CoV-2 viral load	Lessen COVID-19 severity	Downregulated	Wu et al., 2021b
***Bifidobacterium, Roseburium***	Positively correlate with COVID-19 severity	Promote COVID-19 severity	Upregulated	Hazan et al., 2022
***Blautia, Lactobacillus, Ruminococcus***	Positively correlate with proinflammatory cytokines IFN-g, IL-2, IL-4, IL-6, IL-8, IL-10 and TNF-a	Magnify inflammation	Upregulated	Nejadghaderi et al., 2021
***Burkholderia contaminans***	Positively correlate with inflammation biomarkers CRP and IL-6; inversely correlate with the levels of lymphocytes, CD3+ T cells and CD4+ T cells	Magnify inflammation; impair adaptive immune responses	Downregulated	Sun et al., 2022
***Lachnospira, Prevetolla, Roseburia***	Inversely correlate with IL-21	Inhibit inflammation	Downregulated	Khan et al., 2021
***Bifidobacterium adolescentis, Collinsella aerofaciens*,** ***Coprococcus comes, Dorea longicatena, Eubacterium rectale*,** ***Faecalibacterium prausnitzii***	Inversely correlate with CXCL10	Inhibit inflammation	Downregulated	Yeoh et al., 2021
***Citrobacter, Fusobacterium, Peptostreptococcus***	Positively correlate with faecal IL-18 level	Facilitate the cytokine storm	Upregulated	Tao et al., 2020
***Enterococcus faecalis (GroEL)***	Positively correlate with IL-6 and IL-10	Facilitate the cytokine storm	Upregulated	Zhou et al., 2021
***Blautia obeum, Coprococcus catus, Coprococcus comes***	Positively correlate with the number of lymphocytes, CD3+ T cells, CD4+ T cells and CD8+ T cells and lymphocyte proportion	Enhance adaptive immune responses	Upregulated	Xu et al., 2022
***Roseburia intestinalis***	Positively correlate with the number of lymphocytes, CD3+ T cells, CD4+ T cells and CD8+ T cells and lymphocyte proportion	Enhance adaptive immune responses	Downregulated	Xu et al., 2022

## Microbial enhanced intestinal homeostasis

Several metagenomic study outcomes have identified gut dysbiosis as one, among many major risk factors of COVID-19. The pathological imbalance of the microbiota is observed in almost every COVID-19 study case that displayed increased disease progression. Although there has been no significant evidence of a single microbial species affected in the disease associated cases; although groups of associated microbes are strongly believed to be involved in disease development. The antigenic molecules synthesised by the gut microbiota play a pivotal role in protecting host cells from pathogenic invaders so-called ‘omic’ (refers to genomics, transcriptomics, proteomics, or metabolomics) processes. Molecules such as SCFAs are known to be major components that regulate the gut through coordinated regulation of immune cells, gut integrity maintenance and tissue protection. Upon dysregulation of SCFAs, gut integrity is disturbed via enhanced inflammation and the production of cytokines. Various types of SCFA have been identified that work in coordination with host cells, thereby potentiating health outcomes, whilst acting against factors that disrupt gut homeostasis (Xiang and Liu, 2022). During gut dysbiosis, pathobionts (pathogenic microflora) synthesise genotoxins that can cause host cell DNA damage and assist more pathogenic microorganisms in colonizing the gut. For example, the bacterium ‘*E.coli*’ secrete a toxin known as ‘colibactin’ that induces double-stranded DNA breaks and induces mutations that induce chromosomal rearrangements and cell-cycle arrest. In addition, an enterotoxin secreted by the anaerobic gut bacterium ‘*B.fragilis*’ produces an ‘enterotoxin’ that is highly associated with COVID-19. Such toxins dysregulate the gut microbial environment and impair normal gut function. Toxic molecules and microbial metabolites can also invade through cell membranes and reach the brain via the blood circulation; thus, providing a bidirectional link for disease progression, via the provocation of other signalling pathways that contribute an amplifying cascade in the severity of SARS-CoV-2 infection (Quaranta et al., 2022). There is a wealth of evidence that probiotics play significant roles in regulating immune cells and the restoration of the gut microbiome. One such organism that symbiotically produces anti-inflammatory molecules within the gut is *Lactobacillus*. Several such beneficial microorganisms are known to be elevated during COVID-19 infection. Oral administration of probiotics and, the proteomic signatures and the identified biomarkers they provide, pave the way for the development of routine interventions to support clinical decision making, as well as providing hypotheses about COVID-19 disease progression. Discovering more metabolites and protein profiles related to COVID-19 via unbiased omic’s techniques in large patient cohorts and the use of probiotics as inhibitors of disease progression, represents an important future avenue of research. Although enormous efforts have been made to understand the link between COVID-19 and gut microbes, crucial confirmatory evidence remains elusive (Zheng et al., 2020). The output of numerous findings suggests that gut microflora is involved in DNA damage, inflammation, and drug resistance; suggesting that modulation in relative species colonization could act as effective tools in disease prevention/treatment. Recent research outcomes report that the administration of probiotics lessens the effects of diarrhoea and other intestinal symptoms in COVID-19 patients; and are also associated with a reduction of hospitalization rates, and mortality associated with lung failure. Presently, multiple clinical trials are in force to evaluate the effectiveness of probiotics in modifying the gut microbial composition and their potential as adjunctive therapies for COVID-19 patients (Tavakol et al., 2023). Indeed, the abundance of specific microbes can act as clinical biomarkers and entry points for potential therapeutic intervention. Factors limiting the clinical translation of gut microbiome and strategies for resolving current challenges will represent future important research foci.

## Post COVID-19 immune responses of gut microbiota

The connection between gut microbiota and post COVID-19 immunological responses is keenly debated. Although the direct connection between the gut microbiota and post COVID-19 immunological responses is still being investigated, there are numerous potential roles for gut microbiota in recovery and immune control following COVID-19. Due to their confirmed influence on immunological regulation, microbes of gut represent a potential role in assisting the immune system in regaining an appropriate equilibrium. Gut bacteria and microbial metabolites contribute to inflammation reduction. Thus, a healthy gut microbiota may aid in the resolution of inflammation and avoidance of chronic immune activation when the body recovers from the viral induced disease. Additional, immune memory has been related to the gut microbiome and is critical for efficiently responding to future infections or immunisations (Huang et al., 2023). Tissue repair and healing have also been linked to certain gut flora and their metabolites. When lung tissue is damaged as a consequence of COVID-19, a healthy gut microbiota may thus aid in the healing process. Additionally a healthy gut microbiota can improve overall immunological competence and thus avoid opportunistic pathogen colonisation. Some patients have persistent symptoms after COVID-19, a condition termed as “long COVID-19” or PASC. Therefore, gut microbiota relating to immune regulation may have important implications for not only the onset but also the remission of these symptoms (Troisi et al., 2021). Additionally, COVID-19 and its aftermath are stressful. Stress can alter the composition of the gut microbiota, potentially influencing immunological responses and recovery. A healthy gut microbiota may therefore improve stress resilience and immunological modulation. Furthermore, COVID-19 has the potential to influence nutrient absorption and metabolism. Given a healthy gut microbiota is required for optimal nutritional absorption and metabolism, both of which are necessary for immunological function and recovery, it is conceivable an unrecognised feedback axis may also be at play. Nevertheless, the direct correlations between the gut microbiota and post-COVID-19 immune responses need to be thoroughly investigated and validated. Currently, there are ongoing research studies and clinical trials to investigate these interactions, with the aim of discovering potential therapeutic strategies involving the gut microbiota during post-COVID-19 recovery.

## COVID-19 and long-term effects on gut microbiota and immunological effects

The understanding of the post-COVID-19 effects on gut microbiota composition is evolving. However, accumulating research suggests that COVID-19 influences the percentage make-up of gut-living microbes and these effects may be long-lasting. Individuals, who have contracted COVID-19, particularly those expressing severe symptoms, have been reported to exhibit a changed composition of their gut microflora that persists; beyond the point the infection has completely subsided. Some studies strongly suggest a heavy flux in the variety of gut microflora after COVID-19 infection that gradually affects the ability of the gut to perform its functions and is often associated with a loss in microbiota diversity (Banerjee et al., 2023). As COVID-19 can cause a significant and chronic inflammatory reaction throughout the body, the infection may modify the gut environment to favour the growth of particular types of bacteria that affect the overall gut composition. These alterations may be persistent and thus have a long-term impact on intestinal health (Ralli et al., 2023). Additionally, COVID-19 has the potential to cause gastrointestinal symptoms such as diarrhoea and nausea. These symptoms may be connected with changes in the gut microbiota that may linger after the acute symptoms have resolved. Such long-term effects in turn have been shown to alter the immunological response that leads to long-term immune system modifications (Hannoodee and Nasuruddin, 2022). Moreover, COVID-19 is known to have metabolic effects and the availability of gut microbes could play vital roles in related and more general metabolism; thus influencing metabolic health and contributing to the disease’s long-term metabolic repercussions. Therefore, additional future studies will determine the exact processes through which COVID-19 alters the long-term composition of gut microbiota and whether these alterations have associated persistent ramifications. Longitudinal studies that follow the gut microbiota of individuals before, during, and after COVID-19 infection are critical for understanding such long-term impacts.

The relationship between gut microbiota and post-COVID-19 symptoms and immunological dysfunction is still under investigation. Changes in gut microbial diversity may have an impact on immunological responses that cause immune dysfunction in protracted COVID-19. Long COVID-19 is frequently characterised by persistent inflammation and immunological activation. Gut microbiota dysbiosis can lead to chronic inflammation, perhaps aggravating symptoms and contributing to immunological dysfunction. Immune-brain axis communication is characterised by bidirectional signalling that occurs between the gut and the central nervous system. Alterations in the gut microbiota can alter the functioning of this axis and potentially cause neurological symptoms and immunological dysfunction in patients with long-term COVID-19. Dysbiosis may also cause metabolic alterations that affect energy regulation and immunological function. Many long-term COVID-19 patients develop gastrointestinal issues. Alterations in the gut microbiota may contribute to these symptoms and could perpetuate immunological dysfunction (Tirelli et al., 2023). COVID-19 causes the dysregulation of immune signalling molecule expression, such as cytokines. Gut microbiota can regulate cytokine production, and changes in the gut microbiota may contribute to the cytokine imbalances reported in protracted COVID-19 (Low et al., 2023). Changes in the microflora of the gut have systemic implications that extend beyond the gut. Dysbiosis is therefore linked to systemic immunological responses and immune dysfunction across the body. Restoring a balanced gut microbiota may have therapeutic promise for addressing immunological dysfunction and relieving long-term COVID-19 symptoms (Akter et al., 2023). Long COVID-19 symptoms sometimes overlap with other health issues. In these circumstances, the gut microbiota can interact with underlying diseases and potentially worsen immunological dysfunction. It is critical to note that research on the complicated relationship between gut microbiota, post-COVID-19 symptoms, and immunological dysfunction is still ongoing. While there is increasing evidence of correlations, additional research is needed to determine causative ties, mechanisms of action, and viable treatment strategies.

## Post-acute sequelae of SARS-CoV-2 infection

After SARS-CoV-2 infection, numerous individuals have shown persistent “long COVID-19 (PASC)” symptoms. Gut microflora may have a role in the development and duration of PASC symptoms, according to new studies. Some reports have found a shift in the composition of gut microbiota in people with PASC. PASC patients display changes in microbial diversity and a certain abundance of specific bacterial taxa (Gradisteanu Pircalabioru et al., 2022). Individuals with PASC present with lower microbial diversity that may impair the ability of the gut to perform various functions such as immune regulation, nutrient absorption, and metabolism-related problems. PASC is also distinguished by chronic inflammation and decreased immunological activity. Indeed, dysbiosis of the gut microbiota is reported to cause chronic inflammation and immunological dysfunction, thereby worsening PASC symptoms. Some research has found links between particular gut microbiome changes and PASC symptoms. For example, changes in gut flora may be linked to gastrointestinal problems, exhaustion; and cognitive impairment, all of which are prominent in PASC patients. Alterations in gut microbiota can have an impact on the immune-brain axis, perhaps contributing to the neurological symptoms reported in PASC, such as cognitive impairment and mood fluctuations. Changes in the gut microbiota can also affect metabolism; and characterised metabolic abnormalities observed in PASC. Dysbiosis may also cause metabolic changes that affect energy regulation, causing fatigue and other symptoms (Xiong et al., 2023). As gut microbes play a role in regulating immune cells and systemic health, such changes may contribute to the persistence of PASC symptoms long after the virus is eliminated from the body. A more complete understanding of PASC will pave the way for future disease treatment and management strategies. To ameliorate PASC symptoms, interventions focused on the restoration of appropriate gut microbiota, that could involve dietary modifications, prebiotics, probiotics, and faecal microbiota transplantation are an appealing avenue of research (Pantazi et al., 2023). However, such investigations are only in their initial phases. The interconnection between gut microbes and PASC-associated symptoms is complicated and is likely influenced by numerous factors such as differences in microbiota composition, immunological responses, and underlying health issues.

Upon the onset of acute SARS-CoV-2 infection, many patients across the globe develop persistent symptoms that are not resolved over many months (typically classified as symptoms that persist beyond 60 days of SARS-CoV-2 infection). These patients are diagnosed as suffering from long term COVID-19 symptoms or post-acute sequelae of COVID-29 (PASC) and present with symptoms that include fatigue and muscle weakness, sore throat, headache, insomnia, palpitations, chronic rhinitis, chills and dysgeusia. It is important to note that these patients may express disease mechanisms (resulting in PASC symptoms) that are mutually exclusive. Some PASC patients present with symptoms typical of myalgic encephalomyelitis/chronic fatigue syndrome (ME/CFS); a neuroinflammation linked condition with symptoms including musculoskeletal pain and post-exertional malaise (Kedor et al., 2021; Komaroff and Bateman, 2021). PASC symptoms are not surprising, given they are typical of general viral diseases and with frequent exposure to viral and bacterial pathogens (Rasa et al., 2018). Indeed, Enteroviruses acquired through respiratory infection are known to colonize the brain, skeletal muscle and stomach (Chia and Chia, 2008). Furthermore, changes in the gut microbiota and their derived molecular composition are known contributors to ME/CFS (Newberry et al., 2018). Additionally, it is important to note that long term COVID-19/PASC symptoms are also related to associated inflicted organ/tissue injury (Del Rio et al., 2020); for example, the development of lung fibrosis and pulmonary fibrosis (that subsequently causes the deposition of extracellular matrix molecules such as fibronectin, collagen, and laminin in parenchymal lung tissues; hindering gas exchange in the lungs). PASC symptoms also include acute kidney injury and are manifest in reduced glomerular filtration rates (GFR) (Huang et al., 2021). Importantly, it is known that SARS-CoV-2 viruses can persist in certain tissue/organ sites and are not fully eliminated over prolonged periods of time (Vibholm et al., 2021). PASC patients may also present with dysregulated immune cells that cause the reactivation of pathogen reservoirs that can then in-turn infect new cells and elicit PASC-associated, and sometimes severe, symptoms. For example, it is known that several viruses resides inside the human body under dormant, latent, or non-cytolytic forms that upon reactivation can embark upon renewed cycles of infection and disease (Wylie et al., 2014); a so-called ‘multiple-hit-model’.

## Potential techniques to improve post-COVID-19 immune responses

Optimising gut microflora after post-COVID-19 immune response is a promising field of study but specific techniques are still being developed. Restoring the gut microbial balance is frequently a long process that demands long-term dedication. Therefore, the need for consistency in personalised therapies cannot be overstated. Hence, any personalised therapies will require close collaboration with gut health specialists such as gastroenterologists, dietitians, and dedicated medical practitioners (Piccioni et al., 2023). While personalised treatments provide the potential for unique benefits, they also necessitate careful consideration, expert supervision, and continual monitoring. The topic of personalised gut microbiota restoration is fast evolving, but more investigation is needed to properly comprehend the complexities of these techniques. Regarding the diversity of the gut microbiota and its interactions with the immune system, numerous treatment strategies have been posited that support immunological function and recovery from COVID-19. Individual circumstances are likely to differ, and any therapies should be reviewed with healthcare specialists. Indeed, a diet rich in fibre, fruits, vegetables, whole grains, and legumes can stimulate the growth of potentially beneficial gut bacteria that elicit essential anti-inflammatory effects (Mazhar et al., 2023). Prebiotic foods are nondigestible fibres that nourish healthy gut microorganisms. Prebiotics can be found in foods such as garlic, onions, leeks, and bananas. Fermented foods, such as yoghurt, kefir, sauerkraut, kimchi, and kombucha, can also support helpful probiotic bacteria in the gut. Probiotics are living helpful microorganisms that can be taken as supplements. Certain probiotics have been investigated for their ability to improve immune function and gastrointestinal health (Mazziotta et al., 2023). Postbiotics are non-viable microbial products or metabolic by-products of probiotic or prebiotic fermentation. Some postbiotics, such as some specific SCFAs, have been demonstrated to exhibit immune-modulatory properties (Liang and Xing, 2023).

## Potential therapeutic targets and molecular inhibition

The impact of COVID-19 has spread globally and has been associated with an intensive effort to develop therapeutic strategies. Numerous vaccines and therapeutic approaches have already been established and many new interventions have entered clinical trials. The development of vaccines that specifically target SARS-CoV-2 viral proteins to block its reproductive cycle represent on such approach, whereas a second strategy has been focused on host protein synthesis. Although the former vaccine-related approach has encountered limitations associated with the emergence of genetic variants of the SARS-CoV-2 virus (De Clercq and Li, 2016). There are now more than 700 agents designed to functionally impair the SARS-CoV-2 virus in preclinical and/or clinical studies. These target various stages of the virus life cycle; for example, viral spike inhibition, proteolytic inhibition, and RNA synthesis and assembly. To date, some anti-spike monoclonal antibodies (mAbs) have been granted approval, as an early treatment option to out-patients exhibiting preliminary stage symptoms of COVID-19 infection (**Table 4**). However, there is also growing interest in developing polyclonal antibodies based solutions, such as SAB-185, XAV-19, nanobodies of VHH-E, Nb-12, XG014, as well as the biosynthetic protein ensovibep. Additionally, the natural extraction of polyclonal antibodies from the plasma of COVID-19 survivors is considered a potential passive immuno-therapy (Focosi et al., 2022; Senefeld et al., 2022). However, the efficacy of including convalescent plasma as a standard method of therapy poses major challenges that include logistical barriers, limited supply, and antibody dependent triggering of SARS-CoV-2 infection (Lee et al., 2020). The use of anti-spike immunoglobulins are designed to act through two main mechanisms. The first is to target and mask the spike protein and thus prevent viral invasion into the cells (Barnes et al., 2020). The second is to destroy the infecting SARS-CoV-2 virons or virus-infected cells by activating antibody-dependent cytotoxicity or phagocytosis (Lee et al., 2020). Indeed, most of the currently developed antibodies block the interaction of the viral spike protein with the ACE2 receptor. However, developing effective neutralizing antibodies remains highly complex, as the SARS-CoV-2 spike protein undergoes frequent mutations. To counteract this, derived antibodies must specifically target regions within highly conserved non-overlapping epitopes within, or indeed outside, the viral spike protein. However, serum antibody titres decrease over time and can only offer protection for a few months. Hence, the derivation of super-antibodies that provide protection over a long time have been developed through the use of lipid-nanoparticle-encapsulated mRNAs (Deng et al., 2022). In addition to the use of immunoglobulins, small-molecule mediated inhibition of SARS-CoV-2 has been investigated. Molecules such as MU-UNMC-2, P2119, P2165, H69C2, DRI-C23041, and AB-00011778 are currently in progressive clinical trials but will require careful optimization before their approval. Another class of potent inhibitors are represented by the papain like cysteine proteases (PL^pro^). Nearly 30 PL^pro^ inhibitors are reported and are categorized into three classes: i. covalent inhibitors (*e.g.* VIR250 and VIR251) that form C-S thioester linkages, ii. non-covalent inhibitors (*e.g.* GRL0617, F0213, XRS-24, Rac3k, Jun9–72-2, and acriflavine) that block PL^pro^ substrates in the catalytic site; and iii. other non-covalent inhibitors (*e.g.* HE9) that target allosteric binding pockets. PL^pro^ inhibition function by blocking viral derived proteins and facilitate in the restoration of the human immune response. However, choosing the right PL^pro^ is challenging due to the structural similarity to a large family of human deubiquitinating enzymes (DUBs). Although DUB-like proteases are also under investigation as disease targets for other human diseases. Currently none of the described inhibitors have been approved for treatment.

**Table 4:** Clinical trials on the effect of consuming probiotics against COVID-19.

NCT number	Probiotic strain used	Status	Brief summary	Condition	Enrollment	Start & End date	Country
**NCT04756466**	*Lactobacillus* sp.	Unknown	Consumption during COVID-19 severity, and evaluation of immunological effect.	SARS-CoV-2 infection/elderly	201	1/1/2021 - Unknown	Spain
**NCT05043376**	*Streptococcus salivarius* K12	Completed	Adjuvant treatment during mild-to-moderate COVID-19 (Non-ICU) disease.	COVID-19	50	2/4/2022	Pakistan
**NCT05474144**	*Lactobacilli,Bifidobacteria, Akkermansia, Proteobacteria, Firmicutes/Bacteroidetes*	Completed	Monitoring Gut microbiome efficacy through dietary supplements during chronic COVID-19 infection.	COVID-19	83	-/-	Czechia
**NCT04366180**	*Lactobacillus coryniformis* K8	Unknown	Consumption during severity of Covid-19 in health workers.	COVID-19	314	06/2020–10/2020	Spain
**NCT06348212**	*Lactobacillus paracasei* PS23 on	Not yet	Clinical trial to evaluate whether probiotics supplement can improve symptoms of long COVID-19 symptoms.	Long COVID-19	60	04/2024 – 12/2025	Unknown
**NCT04666116**	*Bifidobacterium longum, Bifidobacterium animalis* subsp. *Lactis* and *Lactobacillus rhamnosus*	Unknown	Evaluate the changes occurred after probiotic consumption in post COVID-19 patients	COVID-19	96	4/1/2020–02/2021	Spain
**NCT04517422**	*L. Plantarum* and *P. Acidilactici*	Completed	Clinical research to evaluate how combination effect of probiotics reduce the risk to progress of COVID-19.	SARS-CoV infection	300	2/2/2021	Mexico
**NCT04734886**	*L. reuteri* DSM 17938	Completed	Probiotic response to specific antibodies after COVID-19 infection in healthy adults.	COVID-19	161	-/-	Sweden
**NCT04922918**	*Ligilactobacillus salivarius* MP101	Unknown	Clinical investigation of strains on nasal and faecal inflammatory profiles of COVID-19.	COVID-19	25	7/1/2021	Spain
**NCT05781945**	*Bifidobacterium lactis* LA 304, *Lactobacillus salivarius* LA 302, and *Lactobacillus acidophilus* LA 201	Completed	Clinical evaluation of probiotic-mix (3 strains) in the reduction in faecal calprotectin in patients with COVID-19.	Inflammation	80	3/1/2021–5/1/2022	Italy
**NCT05227170**	*Lactobacillus plantarum* 299v, Lp299v Impact of Lp299v.	Recruiting	Clinical studies of gut microbiome changes strongly associated with Post-Acute Sequelae of SARS-CoV-2 (PASC).	COVID-19	80	-/-	United States
**NCT04907877**	*Bifidobacteria*, and *Lactobacilli* sp.	Completed	Clinical studies in the alleviation of symptoms of acute respiratory tract infections and bursting immune response in COVID-19 out patients.	COVID-19	70	2/7/2021	Brazil
**NCT05175833**	*Streptococcus salivarius* K12, and *Lactobacillus brevis* CD2	completed	Clinical study using an oral gel of probiotic-mix starting in the first ICU day.	COVID-19	70	2/7/2021	Brazil

Potential health risks associated with inappropriate gut microbial composition can be addressed by the addition of probiotics, prebiotics, and FMT. While they have been studied for a variety of illnesses, including gastrointestinal disorders, their use in the context of COVID-19 is still under investigation (Sanekommu et al., 2023). Probiotics comprise health supplements of live bacterial preparations and their composition aims to repopulate the gut and augment patient health and immune response (Mazziotta et al., 2023). Probiotics are defined by their specific strain composition, dosage and the ability of individual patients to retain them. While some probiotics may be healthy, others may be ineffective or even harmful. Prebiotics are non-digestible fibres that encourage the growth and activity of healthy gut bacteria. Prebiotics nourish specific bacteria, particularly those that create anti-inflammatory SCFAs. SCFAs produced by prebiotic-driven bacteria may contribute to immune regulation and inflammation control that in the context of COVID-19-associated inflammation may be advantageous. FMT has also been recommended as a possible strategy for restoring the gut microbial dysbiosis resulting from COVID-19. It has the potential to reduce inflammation and improve the functioning of the gut-lung connective axis. FMT is a challenging procedure with numerous hazards and considerations, such as donor screening, safety, and regulatory issues. Indeed, its efficacy and safety in COVID-19 requires more investigation. These therapies have promise but their precise effects on COVID-19 outcomes are still being assessed. Timing, dosage, individual variance, and the dynamic nature of the gut flora can all have an impact on their efficacy. Furthermore, not all probiotics, prebiotics, or FMT protocols are universally appropriate and interventions should be tailored to the health state and needs of each individual.

## Therapeutic approaches and future directions

The therapeutic potential of modulating gut microbiota to control the COVID-19 disease outcomes has potential. However, microbial-mediated therapy has not yet been effectively launched. Plant based fibres and products (prebiotics) are known to have beneficial effects in balancing the gut microbiome (*e.g.*, Bifidobacterium and Lactobacillus spp). Several plant-based fermented foods are known to lead to the synthesis of beneficial metabolites (*e.g.* SCFAs), contributing to the maintenance of balanced gut microbial populations and gut integrity. Similarly, the oral intake of beneficial microbes as supplements (probiotics) facilitates healthy microbial gut counts (Tieu et al., 2023). Several clinical studies have shown the positive impact of adding healthy microorganisms to the gut and their effectiveness can also be reflected in the lung cells, via the described bidirectional pathway (**Table 5**). For example, the addition of Lactobacillus gasseri sbt 2055 prevents respiratory infection in mouse models. Similarly, administration of Bifidobacterium lactis HN019 to selected study group containing 30 volunteers has also been shown to increase immune response. Post-biotics are microbial products or metabolic by-products created during probiotic or prebiotic fermentation. Some post-biotics, including some SCFAs, have been found to have immune-modulating properties. FMT can be applied to re-establish a balanced gut microbiota through the transfer of faecal material from a healthy donor. While this is an intriguing method, it is still in the early stages of development and should only be used under strict medical supervision (Gibbons et al., 2022). Antibiotics have the potential to disturb the gut flora, hence impairing immune function. Hence, they should be used sparingly and only when absolutely necessary, under the supervision of a healthcare expert, particularly in COVID-19-related cases. Interestingly, a diverse and balanced gut microbiota has been related to regular physical activity and exercise can improve the general health of the immune system. Staying hydrated and eating a well-balanced diet rich in vitamins and minerals will also help improve gut health and immunological function. Individual responses to any gut-modulating methods can differ. Working with gut health specialists can assist in the adaptation of individual therapies and their associated microbiota profiles (Pheeha et al., 2023). Therefore, prior to making any significant changes to dietary food intake, supplementation or health routine, individuals should proceed with caution and contact relevant healthcare experts. The subject of gut microbiota manipulation is complex, yet promising, and research continues.

**Table 5:** Immunoglobulin as therapeutic options for the management of COVID-19.

Drug name	Delivery type	Use	Eligible patients	Resistance likelihood	Status
Inhibitors that block spike-ACE2 interaction					
**Bebtelovimab**	mAb (i.v.)	Tx	Outpatientsb ≤7 days of symptom onset	High (e.g., Omicron)	EUA by the FDA; paused owing to resistance
**Regdanvimab (Regkirona)**	mAb (i.v.)	Tx	Outpatientsb ≤7 days of symptom onset	High (e.g., Omicron, Gamma, Beta)	EUA in many countries; paused owing to resistance
**Sotrovimab**	mAb (i.v.)	Tx	Outpatientsb ≤7 days of symptom onset	High (e.g., Omicron)	Approved or EUA in many countries; paused owing to resistance
**Amubarvimab and** **romlusevimab**	mAbs (i.v.)	Tx	Outpatientsb ≤10 days of symptom onset	High (e.g., Omicron)	Approved in China; discontinued
**Bamlanivimab and** **etesevimab**	mAbs (i.v.)	Tx PEP	Tx Outpatientsb ≤10 days of symptom onset Certain individuals at high risk of COVID-19	High (e.g., Omicron, beta)	EUA in many countries; paused owing to resistance
**Casirivimab and** **imdevimab (REGEN-COV)**	mAbs (i.v. or s.c.)	Tx PEP	Outpatientsb ≤10 days of symptom onset Certain individuals at high risk of COVID-19	High (e.g., Omicron)	EUA in many countries, paused owing to resistance
**Cilgavimab and** **tixagevimab (Evusheld)**	mAbs (i.m.)	PrEP	Certain individuals at high risk of COVID-19	High (e.g., Omicron)	Approved or EUA in many countries, paused owing to resistance

ACE2, angiotensin-converting enzyme 2; COVID-19, coronavirus disease 2019; EUA, emergency use authorization; i.m., intramuscular injection; i.v., intravenous injection; mAb, monoclonal antibody; PEP, post-exposure prophylaxis; PrEP: pre-exposure prophylaxis; Tx, treatment. A High: >10-fold reduction in susceptibility of any SARS-CoV-2 variant. Low: <5-fold reduction in susceptibility.

## Summary

To summarise, COVID-19 is a complex disease with numerous links between various organs, immune cells, secreted molecules, and GIT microbiota. As discussed, the gut microbiota has a well-established influence on immunological function, modulation, inflammatory management, and overall well-being. Indeed, its general significance in regard to human health and disease is becoming more recognised. Understanding how the gut microbiota may be altered and functionally influenced by viruses and pathogenic toxins is a major driver and focus of contemporary research. Specifically, gut dysbiosis, has been linked to numerous health problems in long COVID-19/PASC patients. Although probiotics, prebiotics, and FMT represent some possible therapeutic approaches, via the modulation of the gut microbiota and restoration of immune function, additional clinical investigation is required. Obstacles such as the complexity of the gut microbiota and individual variability, particularly require rigorous clinical trials in order to validate the usefulness of such potential therapies. Indeed, it is increasingly probable that the scope of future research may focus on the potential roles of microbial-derived metabolites; not just in the context of COVID-19 but in a more general sense regarding human health and disease. Notwithstanding, it will be important to precisely elucidate the mechanisms by which the gut microbiota regulates immunological responses, inflammation, and healing following SARS-CoV-2 infection and recovery. Current potential therapies, such as probiotics, prebiotics, and FMT, require significant additional investigation to establish their most optimal application in the context of COVID-19 disease; including determination of the most effective strains, doses, and treatment timing regimes. However, this is not to minimise the findings of current research, as ongoing studies retain the potential to inform treatment decisions, guide therapeutic treatments, and potentially minimise the burden of COVID-19 on healthcare systems across the globe. Robust and interdisciplinary research efforts are required to realise the full potential of leveraging the potential of the gut microbiota to boost immunity and effectively combat COVID-19.

## Author contributions

RM: Writing – original draft, Writing – review & editing. SR: Writing – original draft, Writing – review & editing. AB: Writing – review & editing. MA: Writing – review & editing. HS: Writing – review & editing. SC: Writing – review & editing. MR: Writing – review & editing. KK: Writing – review & editing. HC: Writing – review & editing.
